# Mendelian randomization analysis to analyze the genetic causality between different levels of obesity and different allergic diseases

**DOI:** 10.1186/s12890-023-02636-9

**Published:** 2023-09-18

**Authors:** Yujian Li, Xuan Kan

**Affiliations:** Department of Pediatrics, General Hospital of Tianjin Medical University, No. 154, Anshan Road, Heping District, 300052 Tianjin, China

**Keywords:** Mendelian randomization, Obesity, Allergic disease, Allergic asthma, Causal relationship

## Abstract

**Background:**

The causal relationship between obesity and different allergic diseases remains controversial.

**Methods:**

The Two Sample MR package and Phenoscanner database were used to obtain and filter Genome-Wide Association Study (GWAS) data from the Open GWAS database. Mendelian randomization (MR) analysis was used to study the causal relationship between different levels of obesity and different allergic diseases. The data sets related to obesity and asthma were obtained from the Gene Expression Omnibus (GEO) database. Differentially expressed genes (DEGs) were screened by the limma package. Cluster Profiler and GO plot packages were used for enrichment analysis to verify the results of MR analysis.

**Results:**

Two-sample MR analysis showed a causal relationship between obesity and childhood allergy (age < 16), allergic asthma and atopic dermatitis (*P* < 0.05). In addition, there was also a causal relationship between allergic asthma and obesity (*P* < 0.05), while there was no genetic causal relationship between obesity and allergic rhinitis, eczema, lactose intolerance and so on (*P* > 0.05). Subgroup analysis revealed a causal relationship between both class 1 and class 2 obesity and childhood allergy (age < 16) (*P* < 0.05). Obesity class 1 was associated with allergic asthma, while obesity class 3 was associated with atopic dermatitis (*P* < 0.05). Bioinformatics analysis shows that there were common DEGs between obesity and allergic asthma.

**Conclusion:**

Obesity is a risk factor for childhood allergy (age < 16), allergic asthma and atopic dermatitis, while allergic asthma is also a risk factor for obesity. Class 1 and class 2 obesity are both causally associated with childhood allergy (age < 16). In addition, there is a causal relationship between milder obesity and allergic asthma, while heavier obesity is causally related to atopic dermatitis.

**Supplementary Information:**

The online version contains supplementary material available at 10.1186/s12890-023-02636-9.

Obesity now affects more than 2 billion people worldwide [1], and the COVID-19 pandemic has further increased this figure in recent years [2]. Obesity is classified as obesity class 1, obesity class 2 and obesity class 3 [3]. Previous studies have shown a correlation between obesity and diseases such as diabetes, hypertension, rheumatoid arthritis and fatty liver [4–6]. Allergic diseases include allergic asthma, allergic rhinitis, atopic dermatitis, allergic purpura, allergic conjunctivitis, and lactose intolerance and can occur in children and adults. In addition, allergic diseases affect at least 20% of the global population and their incidence continues to increase annually, affecting both the physical and mental health of patients and imposing a huge economic burden on society and families. Some studies have shown that the total costs associated with allergic asthma in Europe now exceed 19.3 billion euros, in addition to the fact that the proportion of work absences due to allergic asthma reaches twice the proportion of normal employee absences [7–8].

In recent years, the debate and research on the relationship between different levels of obesity and different allergic diseases have increased each year. Several studies have suggested that obesity and allergic asthma may be risk factors for each other, and there may also be a correlation between obesity and allergic rhinitis [9–10]. However, the reliability of the above studies is questionable, large cohort studies have shown that the relationship between obesity and allergic asthma is not clear, probably because the current studies are influenced by a number of confounding factors [11]. In addition, studies on the correlation between different levels of obesity and other allergic diseases such as atopic dermatitis, eczema, allergic urticaria, allergic purpura, allergic conjunctivitis, and lactose intolerance are still relatively scarce. Considering the increasing incidence of obesity and allergic diseases worldwide every year, it is important to clarify whether there is a causal relationship between different levels of obesity and different allergic diseases [12]. Moreover, there is an urgent need to find new epidemiological techniques because traditional epidemiological methods, such as cohort studies, are subject to confounding factors and ethics and thus fail to obtain exact causal relationships [13].

With developments in genomics and genetic epidemiology, numerous genetic variants associated with human diseases have been identified. MR is an epidemiological technique to assess causality through genetic data. As the human genome cannot be changed at will once it is identified, MR analysis greatly reduces the influence of confounding factors, and thus obtains more reliable causal associations [13]. The core of MR analysis is to find instrumental variables (IVs) that can play a connecting role through the 3 core assumptions of IVs, namely, the assumption of association, independence, and exclusivity. The assumption of association is that there should be a strong correlation between the genetic variant and the exposure factor. The assumption of independence means that genetic variation is independent of confounding factors affecting “exposure and outcome”. And the exclusivity assumption requires that genetic variation can only act on outcomes through exposure and not by other means. At the same time, MR analyses need to adhere to the principles of random assignment and free combination in order to minimize the impact of external interventions on the results of the study. For the above reasons, we used MR analysis to analyze the effects of different levels of obesity on different allergic diseases [14].

## Materials and methods

### GWAS data acquisition

**Fig. 1:**
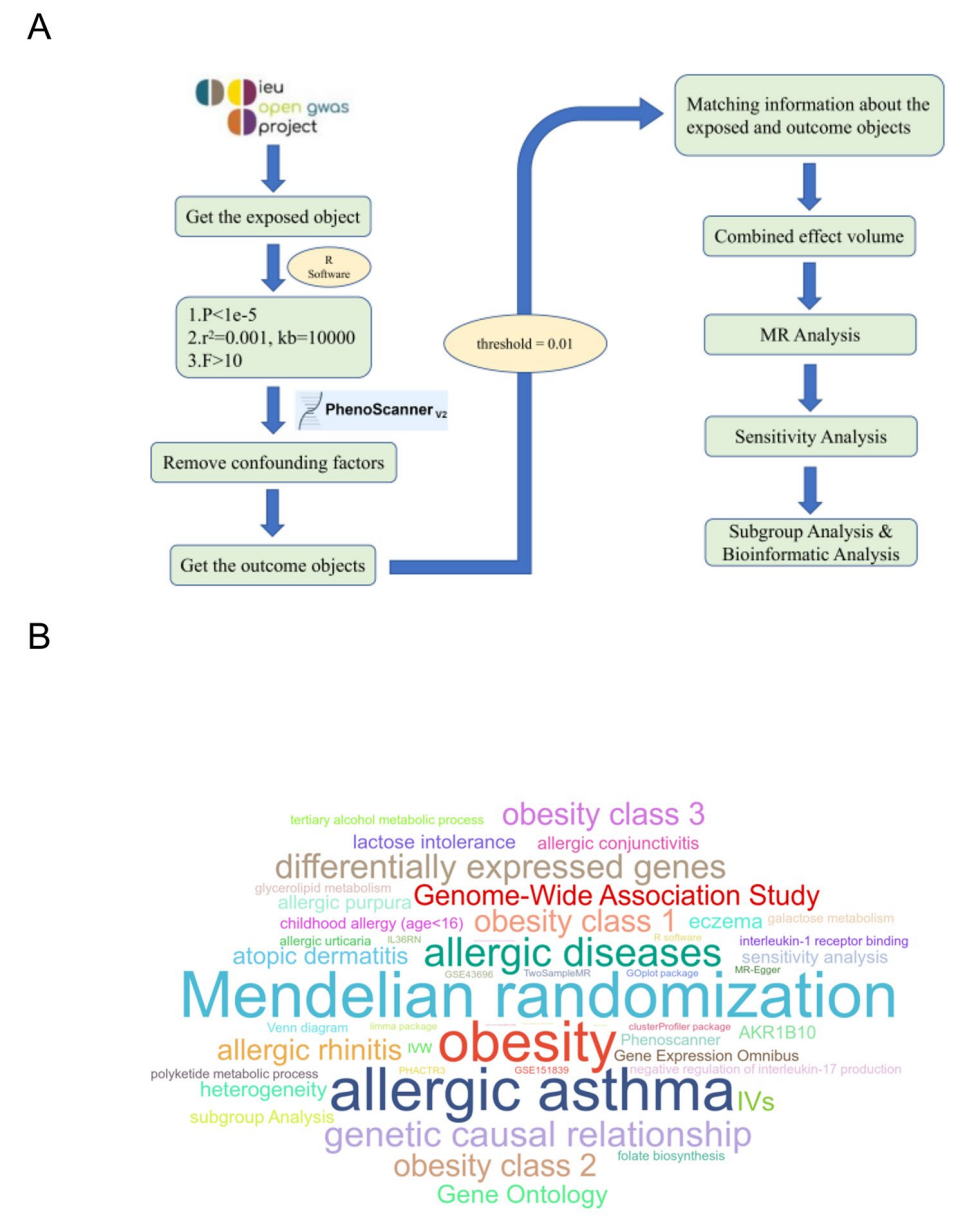
**A**: The flow chart. **B**: The word cloud chart

GWAS is a research method that searches for genetic factors associated with complex diseases by typing large-scale population DNA samples, which can comprehensively reveal the genes involved in the occurrence, development and treatment of diseases. Data for this study were obtained from the OpenGWAS database (https://gwas.mrcieu.ac.uk/). Data from exposed subjects (obesity, childhood allergy (age < 16 years), allergic asthma, atopic dermatitis, obesity class 1, obesity class 2, obesity class 3) and outcome subjects (childhood allergy (age < 16 years), allergic asthma, allergic rhinitis, atopic dermatitis, eczema, allergic urticaria, allergic purpura, allergic conjunctivitis, lactose intolerance, and obesity) were extracted for subsequent analysis with the help of TwoSampleMR package of R software (version 4.3. 0). The flow chart and the word cloud chart are shown in Fig. 1.

### Data screening

IVs were screened for the following conditions: (1) correlation with exposed subjects (*P* < 1e-5) [15]; (2) exclusion of linkage disequilibrium (LD) (r^2^ > 0.001, kb = 10,000) [16]; (3) validation of the strength of association between IVs and exposed subjects (F > 10) [17]; (4) removal of confounding factors using the Phenoscanner database (http://www.phenoscanner.medschl.cam.ac.uk/) [18]. The above conditions will screen for more reliable IVs in this study and thus increase the reliability of the results of this study.

R software was used to merge the data of the exposed and outcome subjects and to match the effect alleles [19].

### MR analysis

MR analysis includes 5 analysis methods, among which IVW and MR-Egger are the most important analysis methods in this study [20]. *P* < 0.05, indicating that exposed subjects affected the outcome subjects.

### Sensitivity analysis

Heterogeneity between each IV was verified using a heterogeneity test. The difference between a fixed effects model and a random effects model is whether an individual variable that does not change over time is correlated with the predicted or independent variable. Fixed effects are more appropriate for examining differences between samples, while random effects are appropriate for inferring overall characteristics from samples. When *P* > 0.05, no heterogeneity was considered to exist, and a fixed-effects model was selected for MR analysis; when *P* < 0.05, heterogeneity was considered to exist, and a random-effects model was selected [21]. The sensitivity of the MR analysis results to individual IV was verified using leave-one-out sensitivity analysis [22].

The presence of horizontal pleiotropy among the multiple IVs was verified using multiple validity test. The biggest difference between the MR-Egger and IVW is whether or not the presence of an intercept term is considered in the regression analysis. The results of IVW analysis were used as the main results of MR analysis when *P* > 0.05 was considered as no horizontal pleiotropy, and the results of MR-Egger analysis were used as the main results of MR analysis when *P* < 0.05 was considered as horizontal pleiotropy [23].

### Subgroup analysis

Obesity was divided into obesity class 1, obesity class 2 and obesity class 3 to perform subgroup analysis. On the one hand, the results of MR analysis were validated, and on the other hand, the causal relationship between different levels of obesity and allergic diseases was further explored.

### Bioinformatic analysis

GSE151839 is a dataset related to obesity obtained from the GEO database (https://www.ncbi.nlm.nih.gov/gds/). GSE43696 is a dataset related to allergic asthma. Differential expression analysis was performed using the limma package to obtain DEGs (|log2(FC)|>1 and *p*.adj < 0.05), and the results were visualized using the ggplot2 package. The results of MR analysis were validated by verifying whether there were common DEGs between DEGs for obesity and DEGs for allergic asthma by means of a Venn diagram. The obtained DEGs were analyzed for Gene Ontology (GO) and Kyoto Encyclopedia of Genes and Genomes (KEGG) enrichment using the clusterProfiler package and GOplot package to understand the main functions and pathways involved in DEGs.

## Results

### Single nucleotide polymorphisms (SNPs)

SNPs are DNA sequence polymorphisms caused by single nucleotide variants at the genomic level and account for more than 90% of known polymorphisms. The most recent and largest sample size of GWAS data was selected for all data in this study. The GWAS ID for obesity was finn-b-E4_OBESITY and contained 218, 735 samples, including 8, 908 in the experimental group and 209, 827 in the control group. This GWAS contained 16, 380, 465 SNPs, and 47 IVs were included after rigorous screening. The GWAS for childhood allergy (age < 16) contained 16, 380, 466 SNPs, and 27 IVs were finally screened. Allergic asthma and atopic dermatitis were screened with 37 IVs and 71 IVs, respectively.

The relevant studies of the outcome subjects were mainly published in 2014–2021, and the study population was from European (Table S1-S2).

### MR analysis

**Fig. 2:**
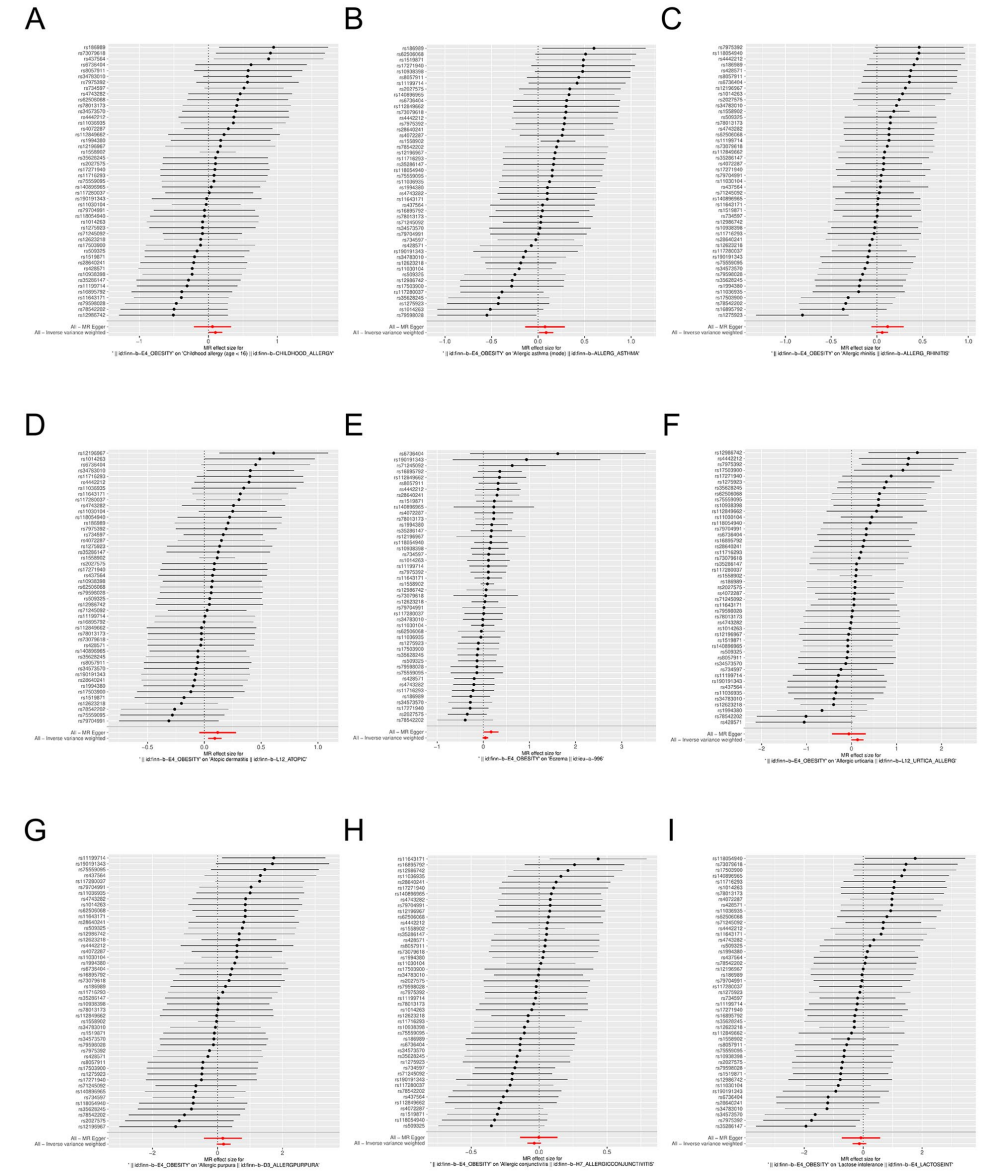
Forest plot for MR analysis of obesity and different allergic diseases. **A**: childhood allergy (age < 16). **B**: allergic asthma. **C**: allergic rhinitis. **D**: atopie dermatit is. **E**: eczema. **F**: allergic urticaria. **G**: allergic purpura. **H** : allergic conjunctivitis. **I**: lactose intolerance. MR: mendel ian random ization

**Fig. 3:**
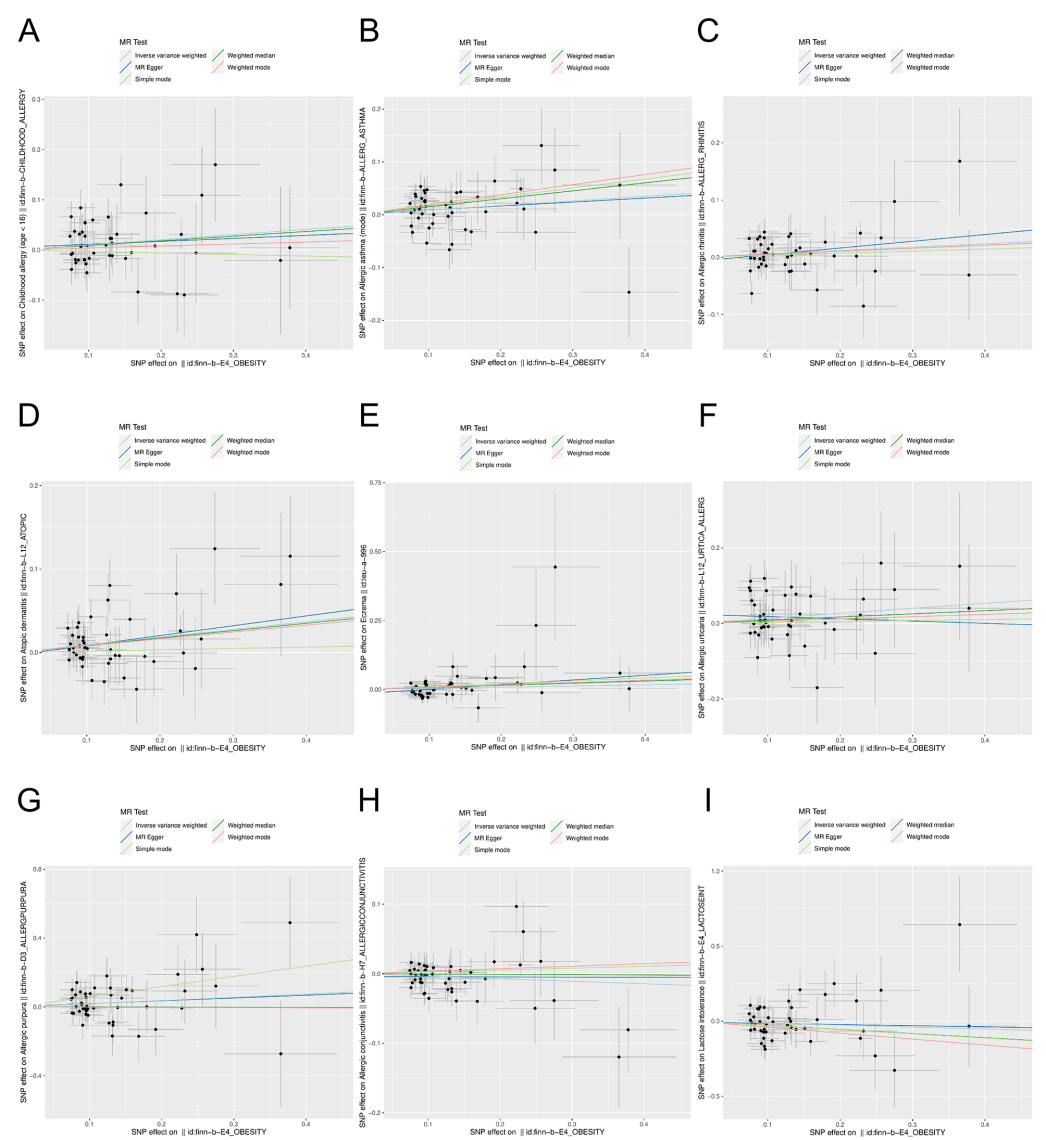
Scatter plot for MR analysis of obesity and different allergic diseases. **A**: childhood allergy (age < 16). **B**: allcrgic asthma. **C**: allcrgic rhinitis. 0: atopic dennatitis. **E**: eczema. **f**: allergic unicaria. **G**: allergic purpura. II: allergic conjunctivitis. **I** : lactose intolerance

**Fig. 4:**
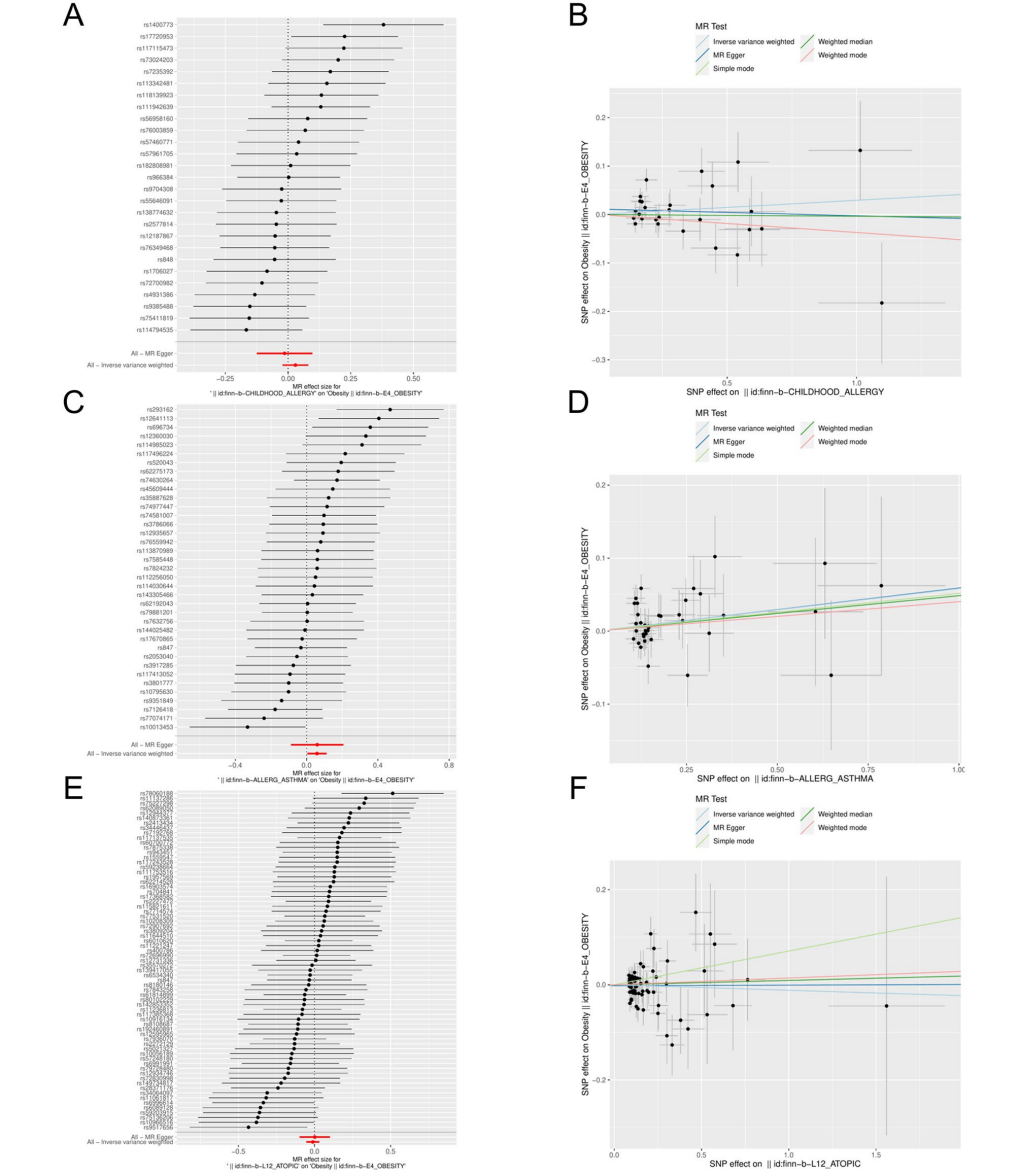
Forest plot and scatter plot for MR analysis of childhood allergy, allergic asthma, atopic dermatitis and obesity. **A**-**B**: childhood allergy (age < 16) and obesity. **C**-**D**: allergic asthma and obesity. **E**-**F**: atopic dermatitis and obesity. MR: mendelian random ization

Two-sample MR analysis showed a causal relationship between obesity and childhood allergy (age < 16) (*P* < 0.05), and obesity was a risk factor for childhood allergy (age < 16) (*OR* = 1.108, *95%CI*: 1.003–1.223). In addition, there was also a causal relationship between obesity and allergic asthma (*OR* = 1.091, *95%CI*: 1.010–1.179) and atopic dermatitis (*OR* = 1.097, *95%CI*: 1.033–1.164) (*P* < 0.05), while there was no genetic causal relationship between obesity and allergic rhinitis, eczema, allergic urticaria, allergic purpura, allergic conjunctivitis, lactose intolerance and so on (*P* > 0.05) (Figs. 2 and 3). Finally, MR analysis was performed with childhood allergy (age < 16), allergic asthma, and atopic dermatitis as the exposed subjects and obesity as the outcome subject. The results showed that there was also a causal relationship between allergic asthma and obesity (*P* < 0.05), and that allergic asthma was also a risk factor for obesity (*OR* = 1.060, *95%CI*: 1.004–1.119) (Fig. 4; Table 1).

**Table 1:** Main results of MR analysis

Outcome	No. SNP	IVW		MR Egger	
		OR(95%CI)	*p*	OR(95%CI)	*p*
Obesity					
Childhood allergy (age < 16)	47	1.108(1.003-1.223)	**0.043**	1.061(0.810-1.389)	0.668
Allergic asthma	47	1.091(1.010-1.179)	**0.027**	1.078(0.871-1.334)	0.493
Allergic rhinitis	47	1.061(0.994-1.132)	0.075	1.125(0.943-1.342)	0.198
Atopic dermatitis	47	1.097(1.033-1.164)	**0.002**	1.125(0.957-1.322)	0.161
Eczema	47	1.044(0.984-1.108)	0.151	1.184(1.007-1.391)	0.047
Allergic urticaria	47	1.141(0.995-1.309)	0.059	0.942(0.649-1.368)	0.756
Allergic purpura	47	1.205(0.972-1.493)	0.088	1.178(0.657-2.113)	0.585
Allergic conjunctivitis	47	0.965(0.917-1.015)	0.162	0.998(0.870-1.145)	0.975
Lactose intolerance	47	0.878(0.694-1.112)	0.282	0.926(0.483-1.774)	0.817
**Childhood allergy (age < 16)**					
Obesity	27	1.030(0.978-1.084)	0.263	0.986(0.883-1.102)	0.808
**Allergic asthma**					
Obesity	37	1.060(1.004-1.119)	**0.035**	1.061(0.916-1.230)	0.436
**Atopic dermatitis**					
Obesity	71	0.989(0.947-1.032)	0.601	1.001(0.906-1.107)	0.980
**Obesity class 1**					
Childhood allergy (age < 16)	41	1.165(1.034-1.313)	**0.012**	1.106(0.805-1.519)	0.538
Allergic asthma	41	1.097(1.004-1.198)	**0.040**	1.045(0.825-1.324)	0.716
Atopic dermatitis	41	1.085(0.996-1.181)	0.060	1.122(0.893-1.409)	0.330
**Obesity class 2**					
Childhood allergy (age < 16)	26	1.110(1.003-1.229)	**0.044**	1.174(0.894-1.541)	0.262
Allergic asthma	26	0.985(0.911-1.066)	0.714	1.057(0.855-1.308)	0.613
Atopic dermatitis	26	1.046(0.983-1.114)	0.155	1.063(0.896-1.260)	0.492
**Obesity class 3**					
Childhood allergy (age < 16)	11	1.090(1.000-1.188)	0.052	0.995(0.745-1.328)	0.971
Allergic asthma	11	1.063(0.983-1.150)	0.128	1.291(1.012-1.645)	0.070
Atopic dermatitis	11	1.064(1.010-1.121)	**0.019**	1.102(0.926-1.312)	0.301

MR: Mendelian randomization; IVW: Inverse variance weighted; No. SNP: number of SNPs

### Sensitivity analysis

The results of the heterogeneity test showed all *P* > 0.05. Therefore, it can be concluded that there was no heterogeneity among the IVs, and the fixed-effects model was finally selected to present the results of the MR analysis (Figure S1, S3). The multiplicity test also showed all *P* > 0.05. Therefore, it was concluded that there was no horizontal multiplicity among the IVs, and the results of the IVW analysis were selected as the main results of the MR analysis (Figure S2-S3, Table 2).

**Table 2:** Sensitivity Analysis

Outcome	Heterogeneity		Pleiotropy	
	Q	*p*	Intercept	*p*
Obesity				
Childhood allergy (age < 16)	43	0.587	0.005	0.738
Allergic asthma	56	0.150	0.002	0.905
Allergic rhinitis	44	0.565	-0.007	0.486
Atopic dermatitis	40	0.735	-0.003	0.742
Eczema	38	0.772	-0.016	0.110
Allergic urticaria	46	0.459	0.024	0.284
Allergic purpura	40	0.720	0.003	0.935
Allergic conjunctivitis	36	0.850	-0.004	0.607
Lactose intolerance	53	0.228	-0.007	0.866
**Childhood allergy (age < 16)**				
Obesity	35	0.102	0.011	0.396
**Allergic asthma**				
Obesity	41	0.245	-0.000	0.990
**Atopic dermatitis**				
Obesity	75	0.258	-0.002	0.783
**Obesity class 1**				
Childhood allergy (age < 16)	31	0.775	0.005	0.728
Allergic asthma	41	0.343	0.005	0.668
Atopic dermatitis	53	0.055	-0.003	0.758
**Obesity class 2**				
Childhood allergy (age < 16)	16	0.873	-0.008	0.672
Allergic asthma	27	0.248	-0.010	0.491
Atopic dermatitis	24	0.413	-0.002	0.849
**Obesity class 3**				
Childhood allergy (age < 16)	8	0.611	0.021	0.532
Allergic asthma	16	0.093	-0.046	0.135
Atopic dermatitis	5	0.898	-0.008	0.689

### Subgroup Analysis

**Fig. 5:**
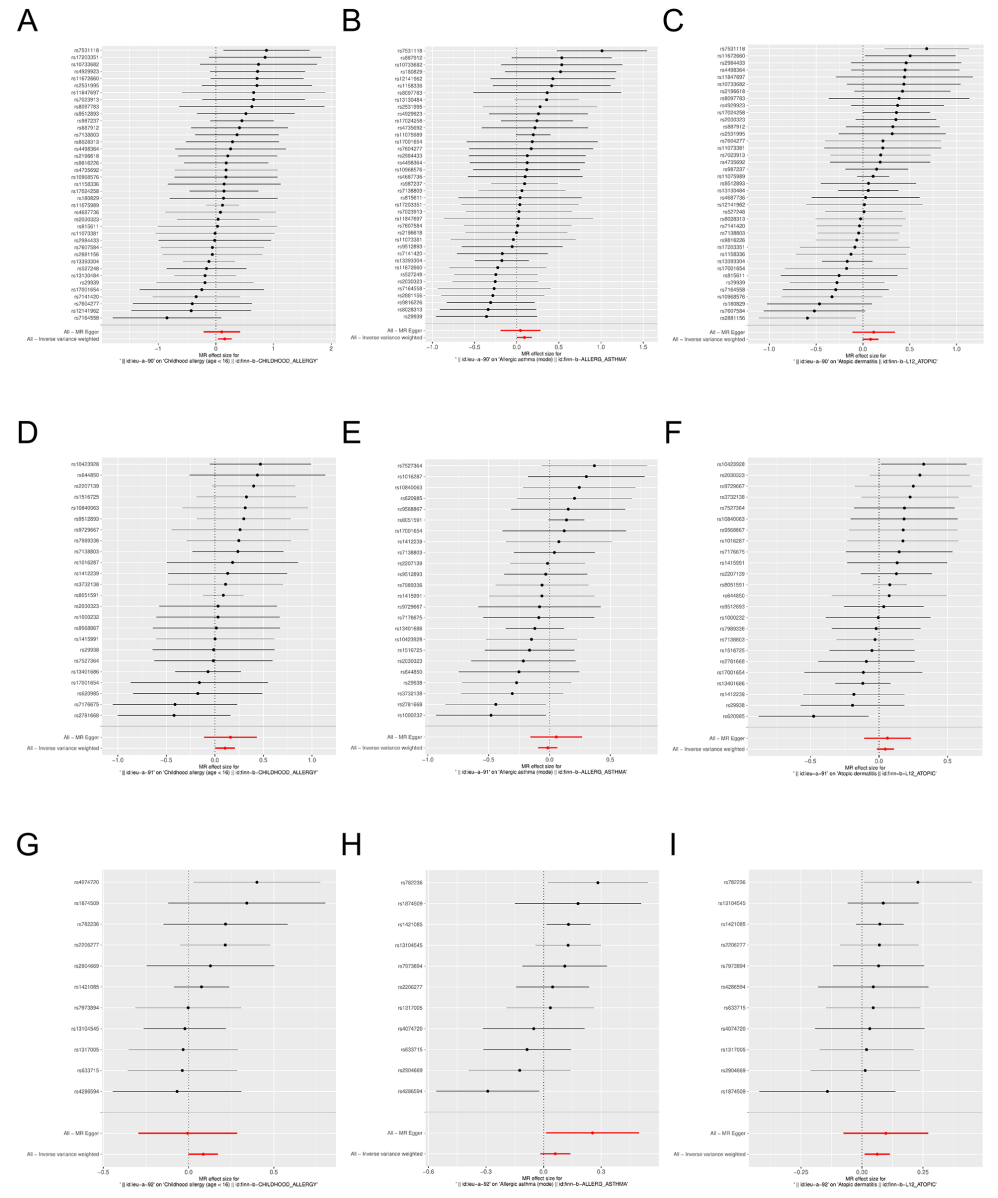
Forest plots for MR analysis of different levels of obesity and different allergic diseases. **A**-**C**: obesity class I and childhood allergy (age < 16), allergic asthm a, atopic dennatitis. **D**-**F**: obesity class 2 and childhood allergy (age < 16), allergic asthma, atopic dermatitis. **G**-**1**: obesity class 3 and childhood allergy (age < 16), allergic asthma, atopic dermatitis

**Fig. 6:**
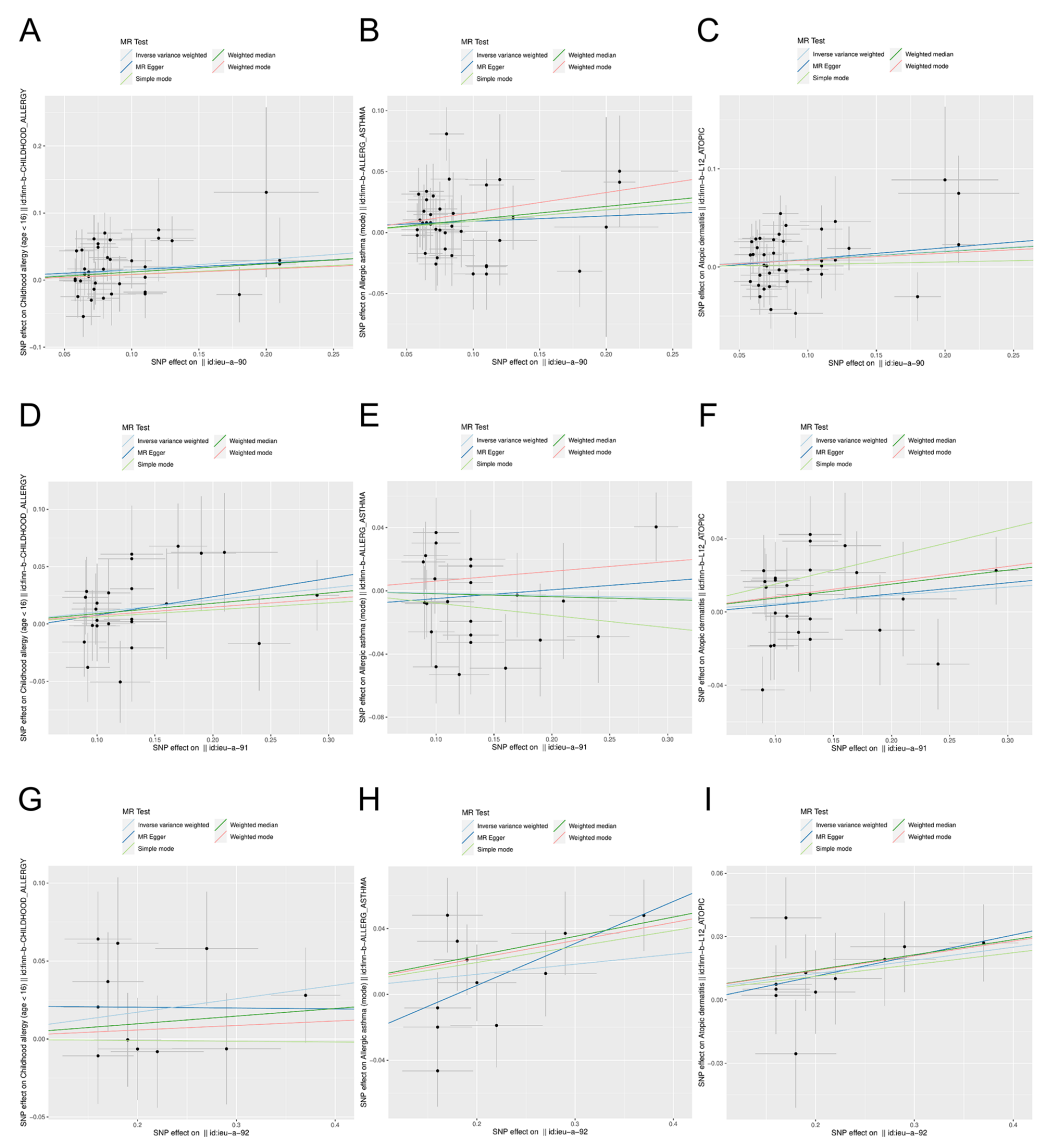
Scatter plots for MR analysis of different levels of obesity and different allergic diseases. **A** -**C**: obesity class I and childhood allergy (age < 16). allergic asthma. atopic dermatitis. **D**-**F**: obesity class 2 and childhood allergy (age < 16), allergic asthma. atopic dermatitis. **G**-**1**:obesity class 3 and childhood allergy (age < 16). allergic asthma, atopic dermatitis

There were 41, 26 and 11 IVs for obesity class 1, obesity class 2 and obesity class 3, respectively (Table S1-S2). Subgroup analysis revealed a causal relationship between both class 1 (*OR* = 1.165, *95%CI*: 1.034–1.313) and class 2 obesity (*OR* = 1.110, *95%CI*: 1.003–1.229) and childhood allergy (age < 16) (*P* < 0.05). In addition, obesity was causally associated with allergic asthma in the milder class 1 obesity (*OR* = 1.097, *95%CI*: 1.004–1.198) and with atopic dermatitis in the more severe class 3 obesity (*OR* = 1.064, *95%CI*: 1.010–1.121) (Figs. 5 and 6; Table 1) (*P* < 0.05). The results of the subgroup analysis were also free of heterogeneity and horizontal pleiotropy (*P* > 0.05) (Figure S4-S5, Table 2).

### Bioinformatic analysis

**Fig. 7:**
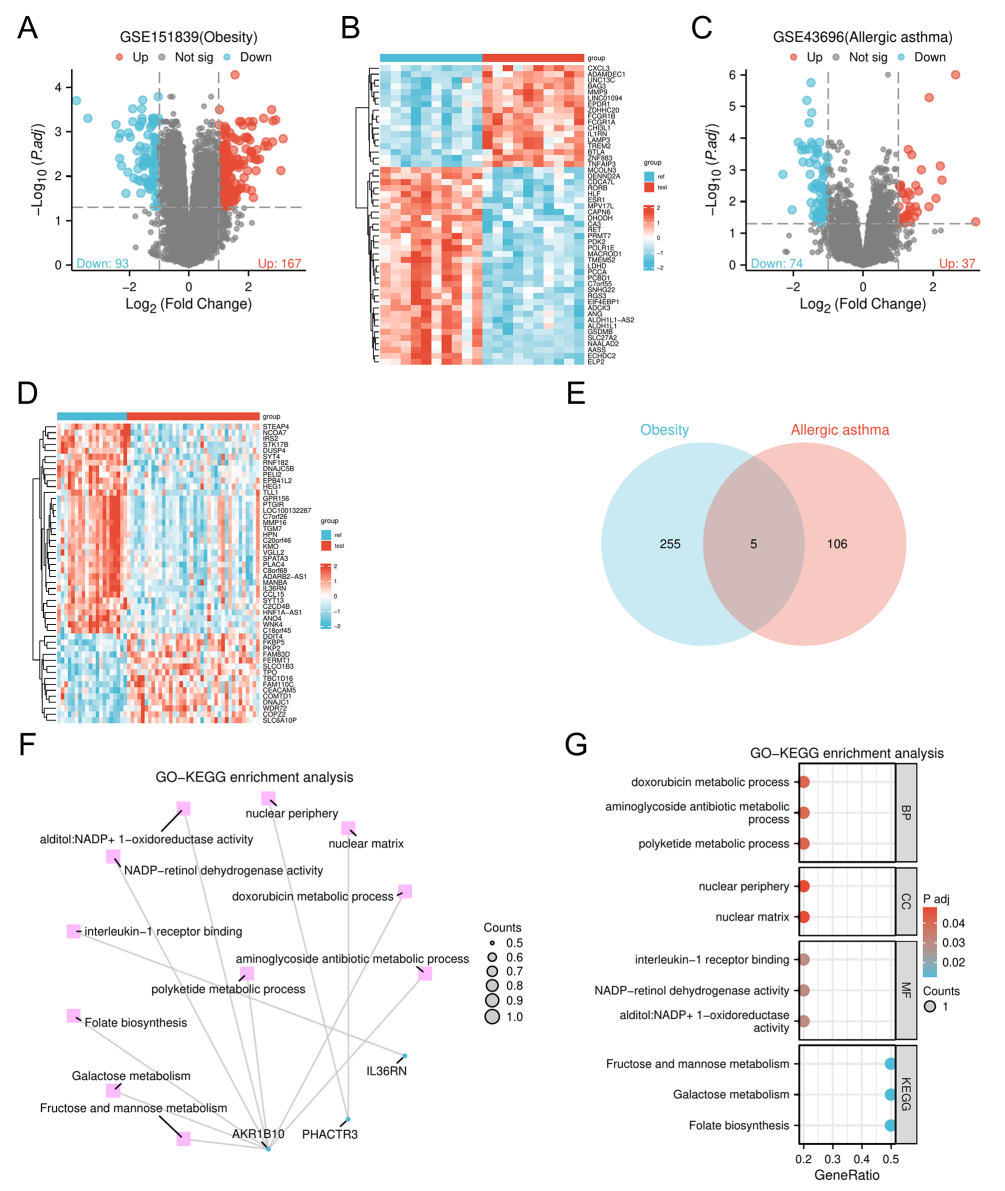
Bioinfonnatic Analysis. **A**-**B**: Volcano map and heat map of obesity-associated DEGs. **C**-**D**: Volcano map and heat map of asthma-associated DEGs. **E** : Venn diagram of DEGs common to obesity and asthma. **F**-**G**: GO-KEGG enrichment analysis of DEGs. DEGs: differentially expressed genes; GO: Gene Ontology; KEGG : Kyoto Encyclopedia of Genes and Genomes

A total of 260 DEGs (|log2(FC)|>1 and *p*.adj < 0.05) were obtained after analysis of the obesity-related dataset GSE151839, of which 167 were highly expressed and 93 were underexpressed (Fig. 7A-B). A total of 111 DEGs (|log2(FC)|>1 and *p*.adj < 0.05) for asthma were obtained, of which 37 were highly expressed and 74 were underexpressed (Fig. 7C-D). The Venn diagram showed that there are indeed common DEGs between obesity and asthma, namely *IL36RN*, *PHACTR3*, *SLAMF7*, *AKR1B10* and *RNF182* (Fig. 7E). These results suggest that the inference that obesity may cause asthma or that asthma may cause obesity is equally supported from a genetic perspective. GO-KEGG enrichment analysis revealed that DEGs common to obesity and allergic asthma mainly have functions such as negative regulation of interleukin-17 production, interleukin-1 receptor binding, tertiary alcohol metabolic process, polyketide metabolic process and so on. In addition, these DEGs are involved in pathways such as folate biosynthesis, galactose metabolism, glycerolipid metabolism and so on (Fig. 7F-G).

## Discussion

The causal relationship between different levels of obesity and different allergic diseases remains controversial. In this study, the causal relationship between different levels of obesity and different allergic diseases was studied by MR analysis, and bioinformatics analysis was used to verify the results of MR analysis. The traditional epidemiological methods, such as cohort studies, are subject to confounding factors and ethics and thus fail to obtain exact causal relationships. At the same time, the results of observational studies may be confounded by reverse causation, where the chronological sequence of the emergence of some of the exposures and outcomes is difficult to discern. Thus, on the one hand, the use of MR analysis compensated for the inability of traditional statistical methods to remove confounding factors, and on the other hand, the validation by bioinformatics analysis further increased the reliability of the results of this study.

Obesity and allergic asthma are risk factors for each other. The two-sample MR analysis in this study showed that obesity is a risk factor for childhood allergy (age < 16), allergic asthma and atopic dermatitis, while allergic asthma is also a risk factor for obesity (*P* < 0.05). There was no genetic causal relationship between obesity and allergic rhinitis, eczema, allergic urticaria, allergic purpura, allergic conjunctivitis, lactose intolerance and so on (*P* > 0.05). Previous studies have pointed out that obesity may contribute to the development of a range of chronic inflammatory conditions in children. The reason may be due to the fact that obesity can lead to dysregulation of the immune system in children by altering the activation status of immune cells [24]. Although obesity has been found to be a common manifestation of asthma in clinical practice, there has been an unending debate about whether obesity causes asthma or asthma causes obesity [25]. This study found that obesity is a risk factor for allergic asthma after better removal of confounding factors by MR analysis, while at the same time allergic asthma is also a risk factor for obesity. Unfortunately, there are still few high-quality studies on allergic asthma causing obesity [9]. Since allergic asthma is mainly associated with Th2 inflammation, some studies have found that obese children may have increased Th2 inflammation and eosinophilia, which This may be one of the associations between obesity and allergic asthma [26–27]. Meta-analysis showed that obesity was positively correlated with allergic asthma and vitamin D deficiency in children, which was also consistent with the results of this study [28]. Atopic dermatitis as an inflammatory skin disease is closely related to the Th2 immune response and microbial homeostasis. Obesity, as a manifestation of metabolic disorder, is associated with gut microbes, which explains why obesity can be a risk factor for atopic dermatitis, consistent with the results of this study [29–30].

Subgroup analyses and bioinformatics analyses similarly supported the association of obesity with allergic diseases, allergic asthma, and atopic dermatitis. Subgroup analysis revealed a causal relationship between both class 1 and class 2 obesity and childhood allergy (age < 16) (*P* < 0.05). In addition, there is a causal relationship between milder obesity and allergic asthma, while heavier obesity is causally related to atopic dermatitis (*P* < 0.05). The results of subgroup analysis to some extent explain why there are some contradictions in previous studies on obesity and allergic diseases [11]. On the one hand, the previous traditional epidemiological studies cannot eliminate confounding factors, but this study uses the method of MR analysis, the causal relationship is more reliable. On the other hand, previous studies have not grouped obese patients into groups to conduct a more in-depth subgroup analysis, so the results may be highly biased. Bioinformatics, a research method that has received considerable attention in recent years, was used for reverse validation of the results of this study. The Venn diagram shows that there were common DEGs between obesity and allergic asthma, which further confirms the results of MR analysis. *IL36RN* is mainly expressed in the esophagus and skin, and its encoded protein is a member of the IL-1 cytokine family, which plays an important role in the occurrence and development of asthma and psoriasis. At the same time, the *IL-36* subfamily participates in the pathogenesis of asthma by secreting cytokines and chemokines for the recruitment and infiltration of T cells, neutrophils and eosinophils [31–32]. *PHACTR3* is mainly expressed in the brain and lung and is associated with the development of lung cancer [33]. *AKR1B10* was found to be involved in signaling pathways in diabetes, obesity and atopic dermatitis, probably because *AKR1B10* is closely associated with the conversion of glycans and lipids in the organism, and in addition is one of the triggers of cytokine storms [34–36]. *SLAMF7* and *RNF182* are related to immune response and protein ubiquitin, respectively. Studies have likewise found a strong association between SLAMF7 and neutropenic asthma and obesity [37, 38]. The study by Sharma V found that obesity may affect the expression of biomarkers of asthma, which in turn promotes asthma onset and progression, consistent with the results of the present study [39]. GO analysis showed that the main functions of the DEGs in common between obesity and allergic asthma were negative regulation of interleukin-17 production, interleukin-1 receptor binding, tertiary alcohol metabolic process, polyketide metabolic process and so on. In addition, these DEGs are involved in pathways such as folate biosynthesis, galactose metabolism, glycerolipid metabolism and so on, which again validated the results of MR analysis in this study.

There are certain strengths and weaknesses of this study. This study is the first to analyze the causal relationship between different levels of obesity and different allergic diseases by MR analysis with the help of GWAS data. We selected the most recent and largest sample size data for inclusion in the follow-up study to ensure the reliability and timeliness of the results. Meanwhile, the MR analysis process in this study was strictly quality controlled according to the 3 hypotheses and sensitivity analysis, thus reducing the influence of confounding factors on the results. The present study had some limitations. First, the samples included in this study were mainly from European, and there may be some geographical selection bias. Therefore, the findings of this study may not necessarily be applicable to Asian ethnic groups. In addition, this study mainly investigated the genetic causality between obesity and allergic diseases, while environment and lifestyle may also have some influence. Finally, to enable rigorous sensitivity analysis, *P* < 1e-5 was chosen as the association threshold for screening IVs in this study. Although the r^2^ and F values were strictly limited, the possibility of false-positive or false-negative results cannot be completely excluded. Considering these, further subgroup analyses in terms of race, age and so on can be attempted in subsequent studies, while high-quality RCT studies can be attempted to obtain more reliable conclusions when conditions permit.

In conclusion, the two-sample MR analysis in this study showed that obesity is a risk factor for childhood allergy (age < 16), allergic asthma and atopic dermatitis, while allergic asthma is also a risk factor for obesity. Class 1 and class 2 obesity are both causally associated with childhood allergy (age < 16). In addition, there is a causal relationship between milder obesity and allergic asthma, while heavier obesity is causally related to atopic dermatitis. Based on the above conclusions, on the one hand, we should strengthen the cooperation between multiple departments and increase the investment in research on the correlation between obesity and allergic diseases. On the other hand, we should strengthen the management of patients with obesity or allergic diseases at an early stage, implement dynamic monitoring and dynamic follow-up. In addition to managing and treating the original disease, it is important to prevent the disease before it occurs, and to prevent changes in the existing disease.

### Electronic supplementary material

Below is the link to the electronic supplementary material.

Supplementary Material 1

Supplementary Material 2

Supplementary Material 3

Supplementary Material 4

Supplementary Material 5

Supplementary Material 6

Supplementary Material 7

## Data Availability

The data sets analyzed during the current study are available in the OpenGWAS database (https://gwas.mrcieu.ac.uk/) (accession nos. finn-b-E4_OBESITY, finn-b-CHILDHOOD_ALLERGY, finn-b-ALLERG_ASTHMA, finn-b-L12_ATOPIC, ieu-a-90, ieu-a-91, ieu-a-92, finn-b-ALLERG_RHINITIS, ieu-a-996, finn-b-L12_URTICA_ALLERG, finn-b-D3_ALLERGPURPURA, finn-b-H7_ALLERGICCONJUNCTIVITIS and finn-b-E4_LACTOSEINT) and GEO database (https://www.ncbi.nlm.nih.gov/gds/) (accession nos. GSE151839 and GSE43696).
