# Supplementary figures and images for: Mendelian randomization analysis to analyze the genetic causality between different levels of obesity and different allergic diseases
Source: BMC Pulm Med. 2023 Sep 18;23:352. doi: 10.1186/s12890-023-02636-9 (PMC10508031; doi:10.1186/s12890-023-02636-9)

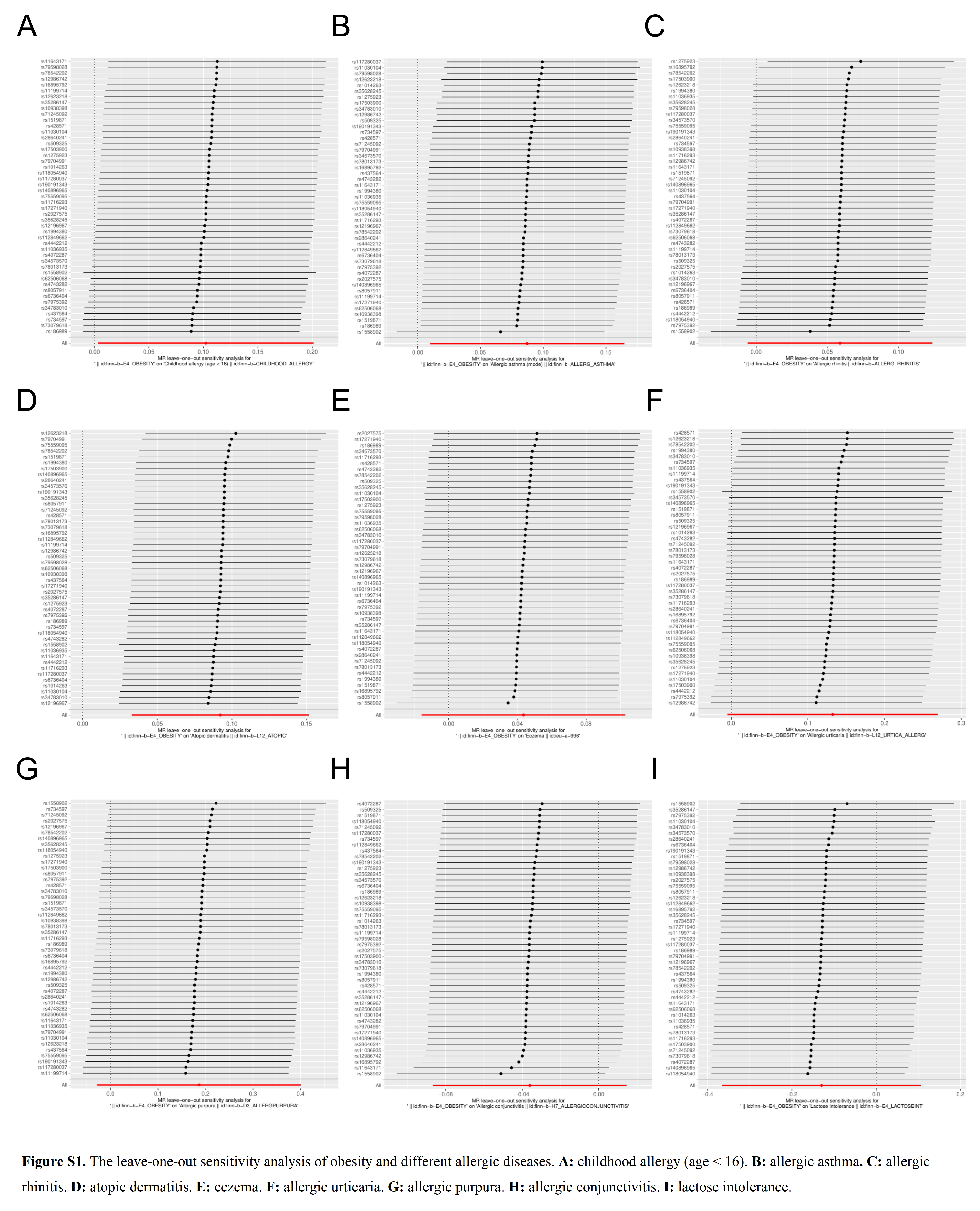

Supplement: Supplementary file 3 — Supplementary Material 3 [file 12890_2023_2636_MOESM3_ESM.png]

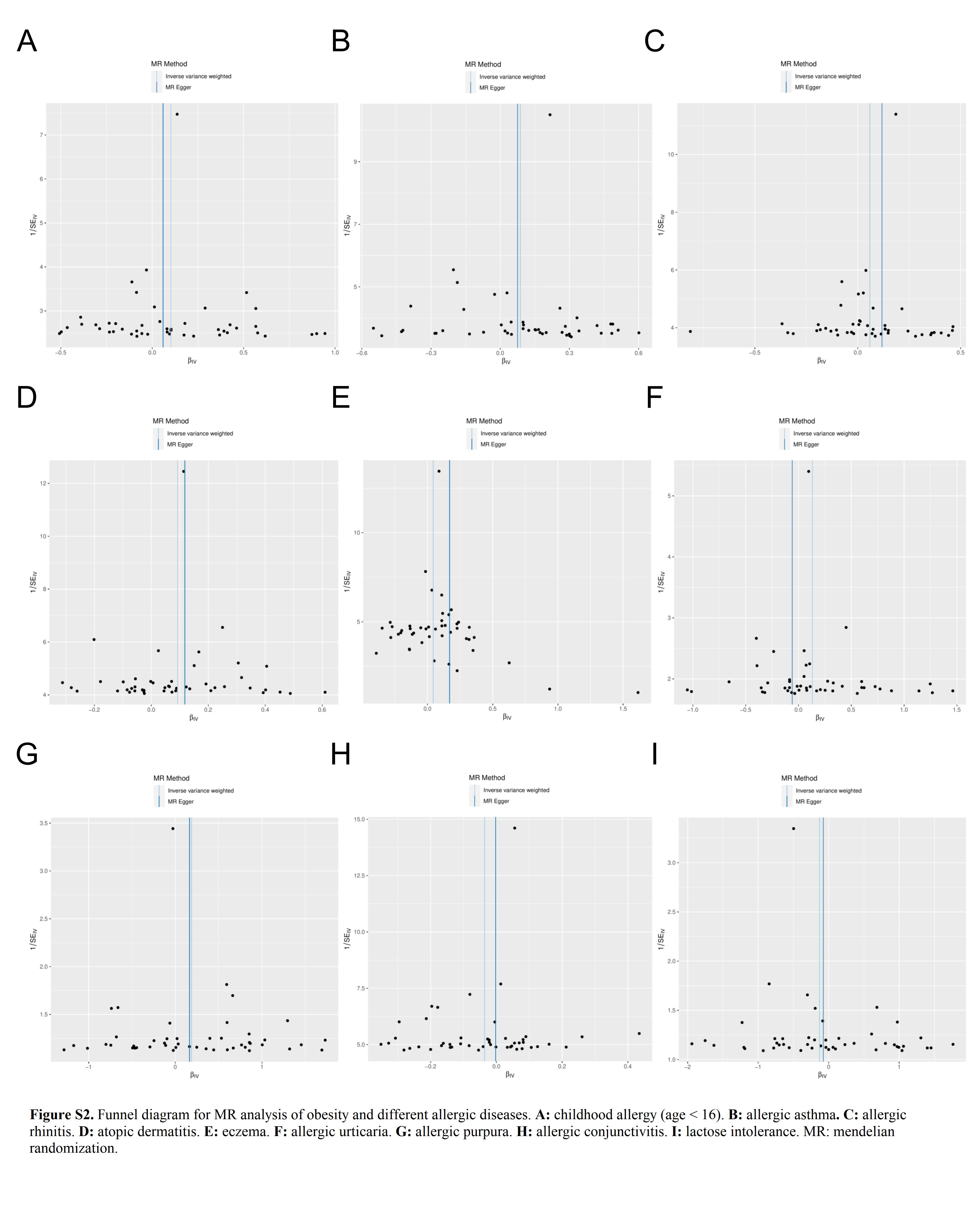

Supplement: Supplementary file 4 — Supplementary Material 4 [file 12890_2023_2636_MOESM4_ESM.png]

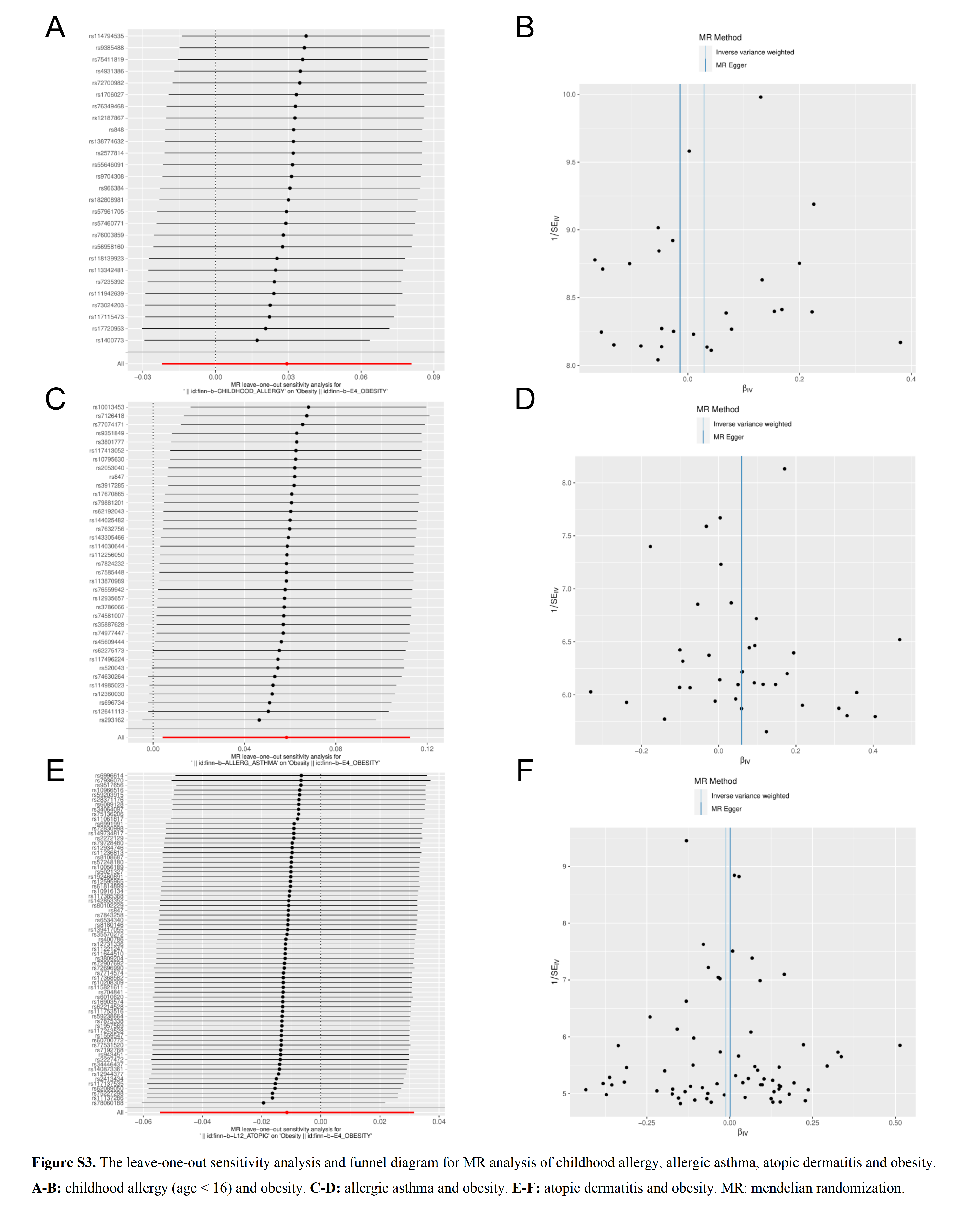

Supplement: Supplementary file 5 — Supplementary Material 5 [file 12890_2023_2636_MOESM5_ESM.png]

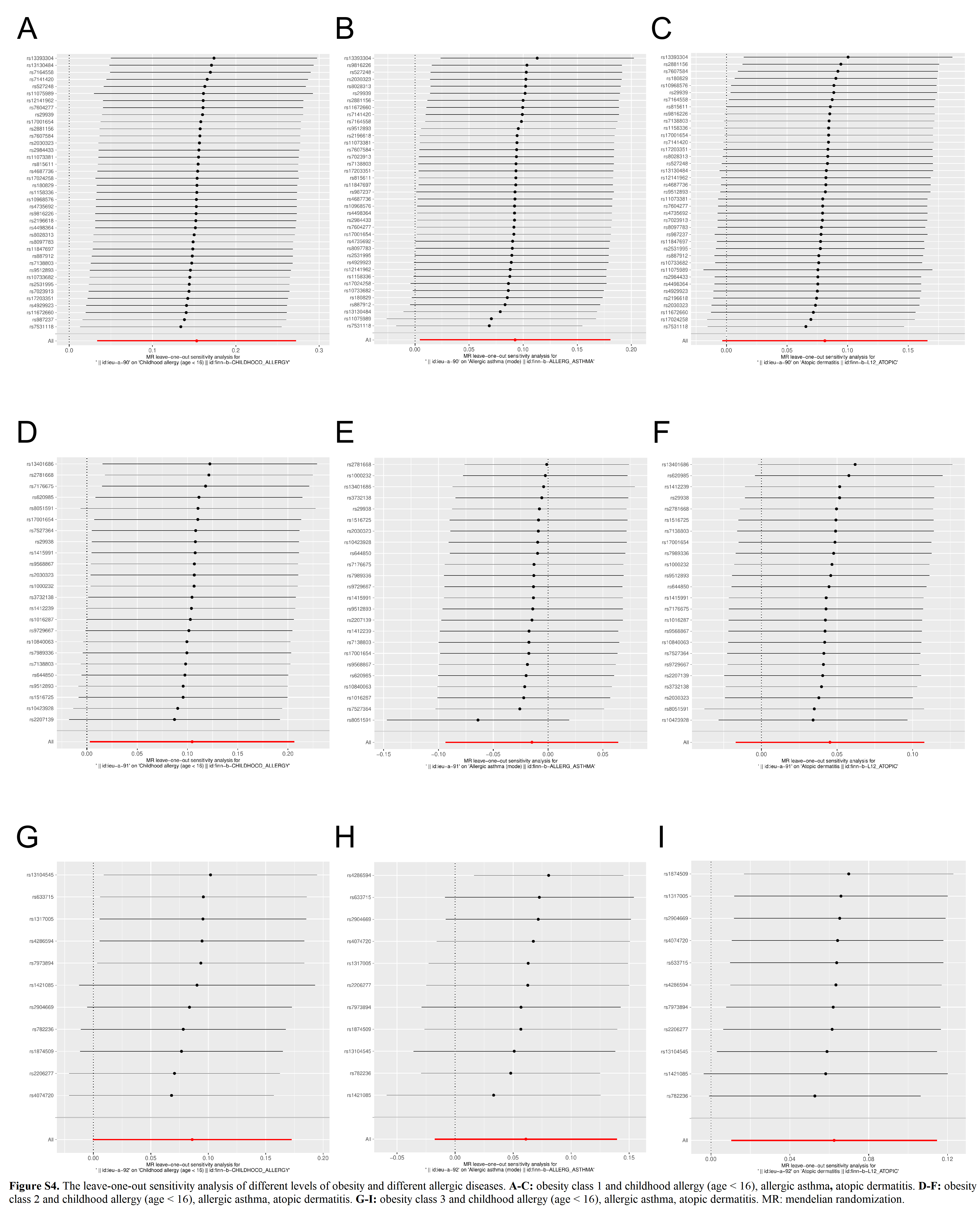

Supplement: Supplementary file 6 — Supplementary Material 6 [file 12890_2023_2636_MOESM6_ESM.png]

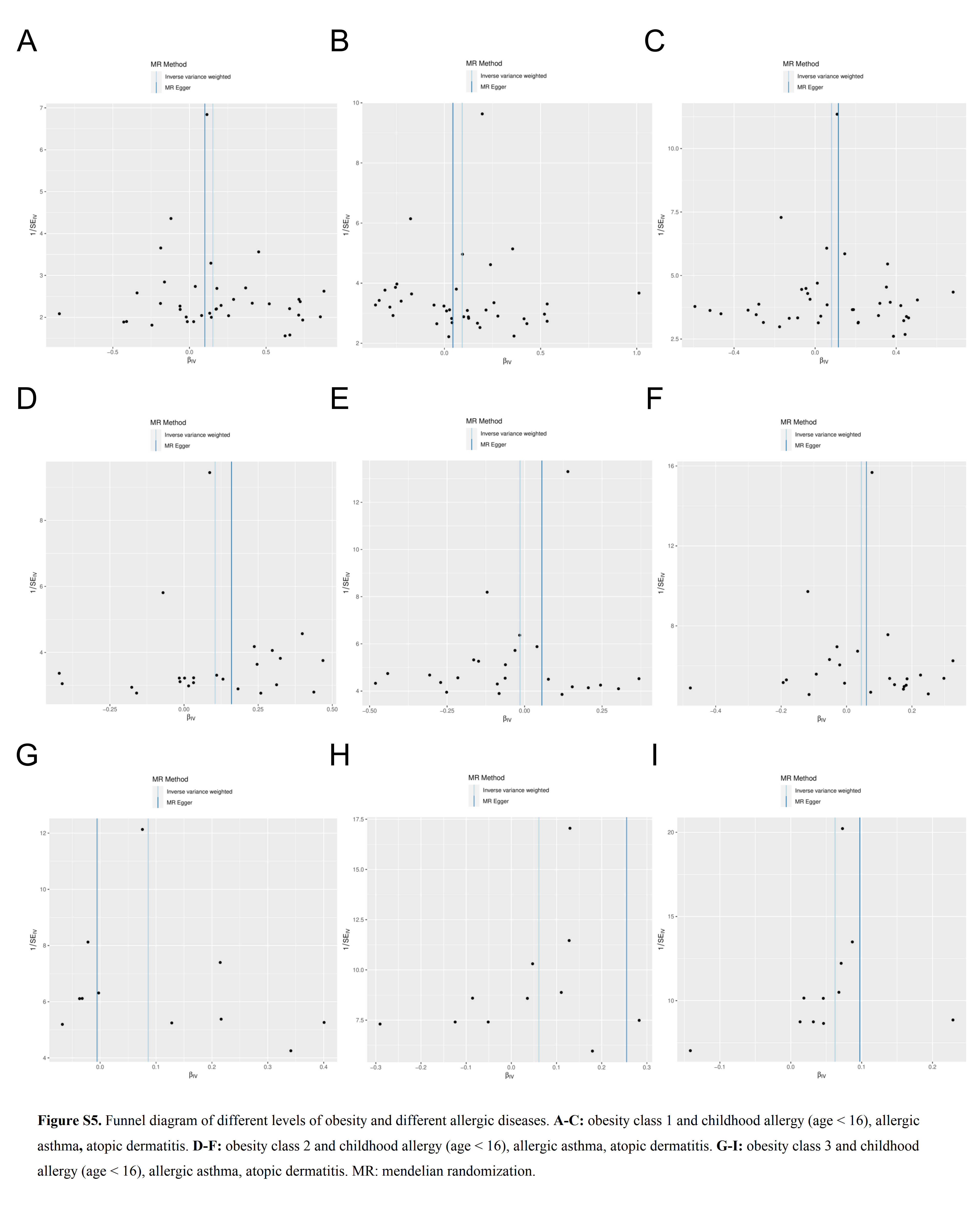

Supplement: Supplementary file 7 — Supplementary Material 7 [file 12890_2023_2636_MOESM7_ESM.png]
